# Supplementary material for: Stakeholder acceptability of the risk reduction behavioural model [RRBM] as an alternative model for adolescent HIV risk reduction and sexual behavior change in Northern Malawi
Source: PLoS One. 2021 Oct 19;16(10):e0258527. doi: 10.1371/journal.pone.0258527 (PMC8525741; doi:10.1371/journal.pone.0258527)

**The RRBM as an alternative model for HIV risk reduction among adolescents**

The Risk reduction behavioral model [RRBM] as furnished to experts for evaluation in the stakeholder acceptability study is an adaptation and expansion on the Comprehensive Sexuality Education (CSE) strategy in adolescent HIV prevention and sexual behaviour change. Also referred to as the abstinence plus approach [8, 15, 20], the CSE strategy advocates the A, B, + C domains of HIV prevention among young people. The A, operationally represents abstinence, B being faithful to one partner and C condom use which comes into play when A cannot be adhered to. Our extensions to the CSE model is premised on proposed 5 levels of associational ABC dynamics and in line with that background how to build adolescent HIV risk reduction and sexual behaviour change. The behavioural categories incorporating ABC associational dynamics were conceptualized in risk-reduction percentile continuum ranging through 0-100 [21].

Specifically, in our conceptualization the risk reduction percentile continuum; 90-100 represented the A function - those who have abstained but also strongly adhere to other risk reduction protocols like HTC, reflecting an extremely high risk reduction percentile [coded 1]. The 80 - 89 percentile represented A & B functions - those who had abstained but were faithful to one partner and also adhered to other risk reduction protocols reflecting high risk reduction percentile [coded 2]. The 60 - 79 percentile represented B & C functions - those who could not abstain, are sexually active but were being faithful to one partner and consistently condomizing reflecting a moderately high risk reduction percentile [coded 3]. The 50 - 69 percentile represented the moderate C function - those who were using condoms with a regular partner but rather entertained other risks like non-regular partners reflecting a moderate risk reduction percentile [code 4]. The 30 - 49 percentile represented the low C function - those who use condoms, might not have a regular partner but also entertained other extreme risk behaviours such as multiple and concurrent partnerships reflecting a low risk reduction or high risk taking percentile [coded 5]. The 0 - 30 percentile represented the risk taking [RT] function - those who engage in risk taking behaviours like unprotected sex, multiple and concurrent partnerships, and transactional sexual intercourse with little or no condom use reflecting an extremely high risk taking percentile [coded 6].

Consistent with the conceptual framework; codes 1 and 2 reflected adaptive behavioural responses; codes 3 and 4 moderate risk reduction behavioural responses and codes 5 and 6 maladaptive behavioural responses. These reflected subsequent codes in analysis- 1, 2 and 3 respectively [1 - adaptive, 2 moderate, 3 maladaptive- risk reduction]. Adaptive and moderate continuums were further classified as composite Adaptive [ code 1] and the maladaptive continuum as composite maladaptive [code 2] -risk reduction versus risk taking respectively. In hypothesis testing therefore, exposure to BCI [Yes or No] would be tested for possible significance on risk reduction [with the aforementioned measures of A- abstinence, B - faithfulness to one partner and C - condom use and other variates like adherence to protocols - HTC, seeking treatment for STIs and male circumcision]. A Matrix for scoring risk reduction was designed to calibrate respondents' categorization into risk reduction percentile levels from 0 - 100. Table 1 below displays proposed participant risk reduction codes, associated behavioural traits, percentiles and coefficients:

Table 1. Participant risk reduction codes, behavioural traits, percentiles and coefficients

| Risk- reduction Codes | Behavioural traits | Percentiles & Coefficients |
| --- | --- | --- |
| Code 1 | [A – function– risk avoidance]  Practicing abstinence and adherence to all other risk avoidance protocols | 90 – 100  (0.90 – 1.0) |
| Code 2 | [ A + B – functions – risk reduction] - Practicing abstinence, being faithful to partner and adherence to risk reduction protocols | 80 -89  (0.80– 0.89) |
| Code 3 | [B + C –functions – risk reduction] -Sexually active, faithful to one partner, consistently using condoms and adherence to risk reduction protocols | 60 -79  (0.60 -0.79) |
| Code 4 | [C – function] - Condom use with regular partner | 50 – 69  (0.50 – 0.69) |
| Code 5 | [Low C – function – risk taking]  Condom use with regular and casual partners | 30- 49  (0.30 -0.49) |
| Code 6 | [High –risk taking] -Risky sexual behaviours, unprotected sex, multiple partners , other risks | 0 - 29  (0.0 – 0.29) |

Risk reduction codes would thus be determined on administered questionnaire responses in the situation analysis and interventional study phases for: sexual experience, ABC self-efficacy, risk perception, risk behaviours likely to drive infection [unprotected sex, transactional sex, multiple and concurrent partnerships, sex under drug and alcohol influence and sex motivated by cultural practices], adherence to biomedical prevention protocols [knowledge, behaviour and behavioural intention on HTC and MMC]. Participant responses and associated scores in addendum would lead to percentile assignments. With that as background in suggesting risk-reduction and sexual behaviour change approaches in line with ABC associational dynamics we therefore went further to propose in our RRBM model incorporation of skills and efficacy building. Skills to include as proposed were practical and coping skills while efficacy building would be centered on both self and social/community efficacy for HIV risk reduction and sexual behaviour change.

Our rational for skills and efficacy building was grounded on their being hallmark for HIV risk reduction among adolescents especially when instilled at an early age during early adolescence [9, 10, 11, 20]. Both self –efficacy and social/community efficacy were included within the backdrop that social determinants of behaviour such as peer and community pressure although often ignored in theoretical frameworks mainly come into the fore in motivating sexual behaviour among sub-Saharan African adolescents [7, 14, 17]. On the other hand self or individual efficacy is critical as it encompasses building the conviction or confidence within adolescents as individuals of executing requisite HIV risk reduction and sexual behaviour change.

Skills inclusion was also premised on the thesis that; decision making, problem solving, assertiveness, as well as self esteem and positive self concept skills building may go a long way in motivating HIV risk reduction. Low self esteem having long been empirically associated with risk taking behaviours, not only with respect to sexual risk taking but other risky behaviours such as drug and alcohol abuse as well as juvenile delinquency [18-21]. We also proposed sexual reproductive health awareness building at an early age as part of our RRBM model. The effectiveness of instilling sexual reproductive health awareness at a tender age is often overlooked with moralistic sexuality education models considering it as likely potentiating sexual immorality among young people [6, 8, 12, 14]. We considered the sexual reproductive health awareness vital to better HIV risk reduction skills and efficacy outcomes and also for broadening the scope beyond only HIV and AIDS to other STIs such as syphilis and gonorrhea as well as for cervical cancer. We also hope some components of the RRBM model could be adapted for Covid 19 prevention, risk reduction skills and efficacy building.

Our justification for instilling sexual reproductive health awareness was further that HIV risk reduction and sexual behaviour change models have mainly been reductionist and limited only to HIV hence our proposal to extrapolate to such other challenges. We also took cognizance within the model of the need to synergize and integrate in programmes and intervention implementation the biomedical prevention strategies of HIV testing and counseling (HTC), Medical Male Circumcision (MMC) as well as sexually transmitted infections (STI) treatment seeking. MMC as a recent approach into HIV prevention not only among young people is proving efficacious [16, 26]. Overall, with the biomedical approaches per se having long been applied separately in Malawi we suggested proposing interventional integration to suit combination options and structural intervention application within social and community contexts.

We further observed that in designing interventions, the structural component might promote targeting of distal correlates such as poverty, gender disparities and culture that may be motivating HIV risk taking especially among adolescent girls [7, 9, 11, 14]. The shift to skills and efficacy building as well as synergizing biomedical strategies with behavioural approaches came against a background of previous emphasis on knowledge building in a majority of behavioural interventions in sub-Saharan Africa. That was despite empirical evidence of HIV knowledge not meaningfully translating into expected behavioural outcomes in many of the interventions [5, 8, 21].

We observed that behaviour change models have been mainly targeting knowledge as a primary outcome and proximate determinant of behaviour change with the hope that such awareness would motivate change in behaviour [5, 12]. The approach has been deemed less effective empirically as such awareness rarely translates to positive HIV risk reduction outcomes and behaviours [10, 11, 14,1 5]. Further, we observed that many previous interventions have not been socially and theoretically contextualized mainly adopting cognitive theoretical frameworks that target individual determinants of sexual behaviour. We noted however through our current research efforts, other systematic reviews and meta-analysis a pointer toward social norms being stronger determinants in sub-Saharan African societies than other parts of the world due to communal as opposed to individualistic socialization [5, 8, 17, 21].

Our model therefore departs from the seemingly status quo by adapting a socio-cultural and ecological framework that proposed peer education within school social contexts in implementation. Our situation analysis [21] and other empirical studies [ 8, 9, 17, 20] suggest peer pressure or what is referred to as the conformity bias as facilitating behaviours driving HIV infection among young people in sub-Saharan Africa and elsewhere. HIV risk behaviours driven by peer conformity include engagement in multiple and concurrent sexual partnerships, unprotected sex, early initiation of sexual activities and sex under drug and alcohol influence [20].

By capitalizing on the same peer social networks that act as key pathways through which high risk sexual behaviours are grounded, we suggested employing peer education in implementing our RRBM intervention. Theoretically we proposed adapting Vygotsky [27] and Bandura’s [28] socio-cultural and social-cognitive theories. The grounds for a socio-cultural or ecological framework were that first, individually based frameworks that apply cognitive behavioural theories have been criticized for overlooking social determinants of sexual behavior [12, 13]. Second, that sexual behavior is determined by multiple factors that transcend individual dynamics, some of which being within cultural, historyed, gendered and structural domains [15]. We also factored in a cognitive behavioural framework to tap on individual dynamics. Constructs from the Theory of Reasoned Action [TRA] [29] were adapted to embrace individual determinants of adolescent sexual behaviour.

The peer education approach in line with social-cognitive, socio-cultural and social learning theories was also premised on learning being guided by older and more knowledgeable significant others or role models in Malawian, sub-Saharan Africa and other communal social context [27, 28]. Knowledge, skills and practices according to these theories are best transferred or learned if guided by more knowledgeable models or social significant -others who are more experienced and can better model and guide younger folks to positive behaviours within their cultural and sociological context . Figure 1 below illustrates the RRBM model in diagrammatic form.

Figure 1. The Risk Reduction Behavioural Model


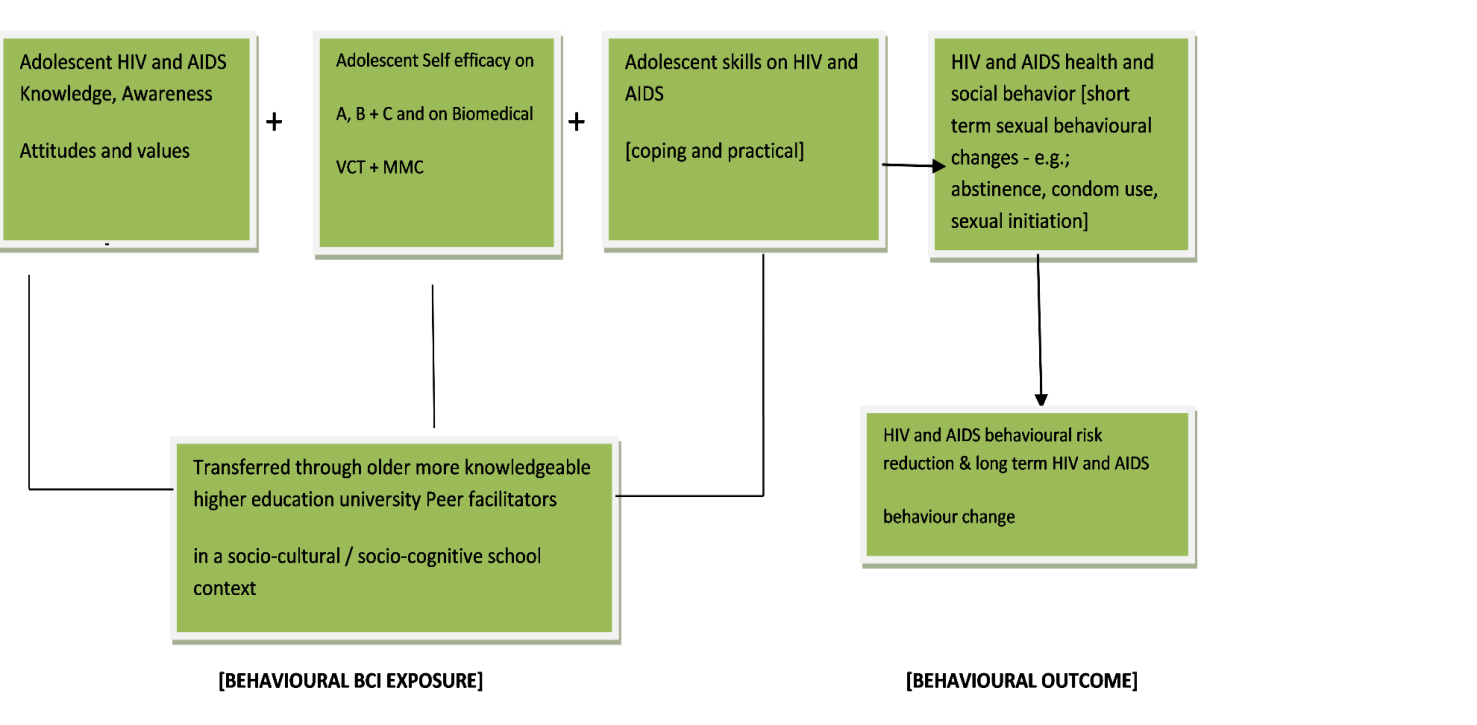

Supplement: S1 File — (DOCX) [file pone.0258527.s001.docx]
