# Supplementary material for: Stakeholder acceptability of the risk reduction behavioural model [RRBM] as an alternative model for adolescent HIV risk reduction and sexual behavior change in Northern Malawi
Source: PLoS One. 2021 Oct 19;16(10):e0258527. doi: 10.1371/journal.pone.0258527 (PMC8525741; doi:10.1371/journal.pone.0258527)
Supplement: S2 File — (DOCX) [file pone.0258527.s002.docx]

**Interview guiding questions**

1] Do you think the model and associated intervention can be extrapolated and potentially scaled up to the wider population of adolescents in Malawi?

2] Compared to standard interventions what is you comparative assessment of the model and associated intervention?

3] Do you think the model and associated intervention can be potentially sustainable?

4] What potential challenges do you anticipate were the model and intervention to be extrapolated and scaled up?

5] What improvements can you recommend on the model and associated intervention to facilitate acceptability and scale up?
