# Supplementary material for: Stakeholder acceptability of the risk reduction behavioural model [RRBM] as an alternative model for adolescent HIV risk reduction and sexual behavior change in Northern Malawi
Source: PLoS One. 2021 Oct 19;16(10):e0258527. doi: 10.1371/journal.pone.0258527 (PMC8525741; doi:10.1371/journal.pone.0258527)
Supplement: Attachment — Submitted filename: PLOS One_ Responses to reviewers.docx [file pone.0258527.s003.docx]

Dear Editor/(s)

Thank you for the decision for us to revise our article # PONE-D-20-17629 – title, ‘Stakeholder acceptability of the risk reduction behavioural model [RRBM] as an alternative model for adolescent HIV risk reduction and sexual behaviour change in Northern Malawi’.

We have compiled our revisions beginning with those associated with PLOS One requirements and followed by revisions on queries raised by reviewers. In our rebuttal we first outline the query raised by either editor or reviewer and present its associated response as follows:

**Revisions – Editorial PLOS One requirements**

1. **Query** – Please ensure that your manuscript meets PLOS ONE’S style requirements including those for file naming:

**Response** – Formatting of manuscript main body, files, title and author affiliations done in line with PLOS One guidelines.

2. **Query** - When reporting the results of qualitative research, we suggest consulting the COREQ guideline. Moreover, please provide the interview guide used

**Response** – COREQ guidelines for reporting qualitative results have been adhered to in line with - [Tong A, Sainsbury P & Craig J. Consolidated criteria for reporting qualitative research (COREQ): a 32-item checklist for interviews and focus groups. International Journal for Quality in Health Care, 2007; 19(6): 349 – 357]. The interview guide has been included as a supporting file – SI File. File 1 Title- Interview guide.

\

3. **Query** - In your Data Availability statement, you have not specified where the minimal data set underlying the results described in your manuscript can be found …..

**Response** – Minimal data set has been deposited in draft with Qualitative Data Repository (QDR) – University of Syracuse, and can be accessed at <https://doi.org.10.5064/F6RGY6T3>

4. **Query** – We note that you have provided funding information that is not currently declared in your Funding Statement. However, funding information should not appear in the Acknowledgements section or other areas of your manuscript. We will only publish funding information present in the Funding section of the online submission form.

Please remove any funding-related text from the manuscript and let us know how you would like to update your Funding Statement.

Please include your amended statement within your cover letter; we will change the online submission form on your behalf.

**Response**- Funding related text has been removed from any section in the manuscript and has been instead included in the cover letter.

5. **Query** – Your ethics statement should only appear in the Methods section of your manuscript. If your ethics statement is written in any section besides the Methods, please delete it from any other section.

**Response** – The ethics statement has been included only in the Methods section and removed from declarations section with the entire declarations section removed.

**Revisions – Reviewer comments**

**Introduction**

1. **Query** - It is unclear what the RRBM is and how it differs from the RRBI. Describe the components of the intervention, the duration of the intervention, the target population, and the specific behaviours the intervention is meant to address.

**Response** – The RRBM intervention has been presented as a separate sub-section in the methods with title, ‘The RRBM as an alternative model for HIV risk reduction among adolescents’ - (pages: 10-17)

Components of the intervention as it was tested for efficacy, the duration of the intervention, the target population and the specific behaviours model was meant to address are presented in another sub-section of the methods, title – ‘The RRBM model as tested for efficacy through a peer education intervention with adolescent participants in Northern Malawi’ - (pages: 18-19)

2. **Query** – Examining triability and feasibility were considered part of the purpose of the study but this was not explored in the methods, results or discussion. The article would be strengthened by grounding the article in concepts from ‘Implementation science’. Consider using Proctor E et al (2011).

**Response** – As recommended and in line with concepts from ‘Implementation science’ – Proctor et al (2011); the concepts triability and feasibility have been removed from manuscript - (pages: 2 & 3) and the same also applies to replacing the concept fidelity with the concept effectiveness in the results section (page – 22).

**Methods**

3. **Query** – Page 6 – The justification for selecting individual interviews over group interviews /workshop does not need to included. Cut the following sentence, ‘The choice of the individualized process compared …….’

**Response** – The sentence per se has been removed as recommended (page 6).

4. **Query** – Page 6 – Provide more details about the interview process. Where were interviews conducted? Who conducted the interviews? Approximately how did they last?

**Response** – Details about the interview process have been presented as recommended; including where interviews were conducted, the interviewer and duration (page 7)

5. **Query** – Page 7 - Provide more information about the data analysis process. How many people were involved in analysis? How did they reach consensus? Was analysis software used?

**Response** – As recommended, information about the data analysis process; including number of people involved, how consensus was reached, and whether software was used has been presented on (page 8).

**Results**

6. **Query** – Page 10 – The quotes and associated text for Theme 2 do not address implementation fidelity as defined in Rabin et al (2008).

**Response** – The concept ‘fidelity’ has been replaced by the concept ‘effectiveness’ in associated text for Theme 2 to align with intended objective (page 22 & 32).

7. **Query** – The quotes about the intervention needing to meet the needs of rural and out of school girls and girls with disabilities is closer to the implementation outcome of ‘reach’. Authors need to describe how teacher conservatism can affect fidelity.

**Response** – Point was taken and addressed with the replacement of the concept fidelity with the concept effectiveness as highlighted in 6 above.

8. **Query** – Page 12 – Provide more of description of ‘youth priorities and needs’. The quotes included in the table aren’t all aligned with that theme for example, the quote, ‘focus in implementation should be on already existing structures in communities but perhaps lacking empowerment and capacity building’ doesn’t seem like a youth priority or need.

**Response** – The quote and other sections have been qualified with the concept youth as intended where necessary to align with the theme and objective and revised as follows:

‘focus in implementation should be on already existing youth structures in communities but perhaps lacking youth empowerment and capacity building’ (page 24 & 25).

9. **Query** – The last two quotes in the table more address the theme of sustainability in Table 5.

**Response** – The quotes per se have been moved to the theme of sustainability in previously Table 5, but now Table 6 (page 29).

**Discussion**

10. **Query** – Page 18 – Paragraphs two and three discuss structural and combination intervention approaches, however that was minimally discussed in the results section. Only one quote in Table 2 describes the need for structural interventions. Cut those paragraphs.

**Response** – The paragraphs on structural and combination interventions have been removed as recommended (page 30 – highlighted).

11. **Query** – The first strength that this study is one of the few studies that include stakeholder involvement can be tampered a little bit. There are many studies that involve stakeholder interviews.

**Response** – The statement has been revisited as follows: ‘Our study is one among intervention studies with a component involving seeking input from stakeholders …’ (page 33 - highlighted)

We hope the responses cover all the queries raised in the review.

Regards,

Marisen Mwale
