# Supplementary material for: Stakeholder acceptability of the risk reduction behavioural model [RRBM] as an alternative model for adolescent HIV risk reduction and sexual behavior change in Northern Malawi
Source: PLoS One. 2021 Oct 19;16(10):e0258527. doi: 10.1371/journal.pone.0258527 (PMC8525741; doi:10.1371/journal.pone.0258527)
Supplement: Attachment — Submitted filename: PLOS One_ Responses to reviewers.docx [file pone.0258527.s004.docx]

Dear Editor/(s)

Thank you for the decision for us to revise our article # PONE-D-20-17629R1 – title, ‘Stakeholder acceptability of the risk reduction behavioural model [RRBM] as an alternative model for adolescent HIV risk reduction and sexual behaviour change in Northern Malawi’.

We have compiled our revisions beginning with those associated with PLOS One requirements and followed by revisions on queries raised by reviewers. In our rebuttal we first outline the query raised by either editor or reviewer and present its associated response as follows:

**Revisions – Editorial PLOS One requirements**

1. **Query** – Please review your references list to ensure that it is complete and correct. If you have cited papers that have been retracted, please include the rationale for doing so in the manuscript text, or remove these references and replace them with relevant current references. Any changes in the reference list should be mentioned in the rebuttal letter that accompanies your revised manuscript.

**Response** – References have been revised as recommended. Reference 61 has been swapped with reference 26 to suit the link of socio-ecological and socio-cultural models (citations 26, 27 and 28) as adapted in the paper (page 40).

2. **Query** - The authors have sufficiently responded to the reviewer comments. However, the section ‘The RRBM as an alternative model …’ is too long and detracts from the overall manuscript. Reviewer # 1 has suggested cutting this section. As an alternative, the authors could reduce this to a single paragraph and include the remainder as a supplement. This would significantly improve the manuscript focus and flow.

**Response** – As recommended by the reviewer, the section ‘The RRBM as an alternative model ….’ Has been cut and the content has been retained as an additional supplementary file 1 (S1 File 1. Title – The Risk reduction behavioural model).

**Revisions – Reviewer comments**

1. **Query** – The manuscript is much improved and the majority of my comments have been addressed. One area that needs to be revised is in the description of the RRBM. While it was helpful to read a description, it was quiet long. The section titled, ‘The RRBM model as tested for efficacy through a peer-education intervention with adolescent participants in Northern Malawi’ provides sufficient detail. Rename that section ‘The Risk Reduction Behavioural Model.’ Cut the section ‘The RRBM as an alternative model for HIV risk reduction among adolescents’.

**Response –** As recommended by Reviewer # 2, the section titled ‘The RRBM model as tested for efficacy through a peer-education intervention with adolescent participants in Northern Malawi’ has been renamed to ‘The Risk Reduction Behavioural Model’ with a bit of revisions (pages 10 - 11).

Additionally the section ‘The RRBM as an alternative model for HIV risk reduction among adolescents’ has been cut as recommended and the content thereof included as a detailed description of the risk reduction model in supplementary file 1 (S1 File 1. Title – The risk reduction behavioural model).

We hope the responses cover all the queries raised in the review.

Regards,

Marisen Mwale
